# The Endolysosomal Transporter DMT1 is Required for Morphine Regulation of Neuronal Ferritin Heavy Chain

**DOI:** 10.1007/s11481-023-10082-x

**Published:** 2023-09-04

**Authors:** Elena Irollo, Bradley Nash, Jared Luchetta, Renato Brandimarti, Olimpia Meucci

**Affiliations:** 1 Department of Pharmacology & Physiology, Drexel University College of Medicine, 245 North 15th Street, Philadelphia, PA 19102 USA; 2 Department of Pharmacy and Biotechnology, University of Bologna, Via Marsala, 49, Bologna, BO 40126 Italy; 3 Department of Microbiology & Immunology, Drexel University College of Medicine, 245 North 15th Street, Philadelphia, PA 19102 USA; 4 Center for Neuroimmunology & CNS Therapeutics, Institute for Molecular Medicine & Infectious Disease, Drexel University College of Medicine, 245 N. 15th Street, Philadelphia, PA 19102 USA

**Keywords:** DMT1, SLC11A2, Iron, Neuron, Morphine, neuroHIV

## Abstract

NeuroHIV and other neurologic disorders present with altered iron metabolism in central nervous system neurons. Many people with HIV also use opioids, which can worsen neuroHIV symptoms by further dysregulating neuronal iron metabolism. Our previous work demonstrated that the μ-opioid agonist morphine causes neuronal endolysosomes to release their iron stores, and neurons respond by upregulating ferritin heavy chain (FHC), an iron storage protein associated with cognitive impairment in neuroHIV. Here, we investigated if this process required divalent metal transporter 1 (DMT1), a well-known iron transporter expressed on endolysosomes. We first optimized conditions to detect DMT1 isoforms (DMT1 1B ± iron responsive element) using fluorescently labeled rat DMT1 constructs expressed in HEK-293 cells. We also expressed these constructs in primary rat cortical neurons to compare their expression and subcellular distribution with endogenous DMT1 isoforms. We found endogenous DMT1 isoforms in the cytoplasm that colocalized with lysosomal-associated protein 1 (LAMP1), a marker of endolysosomes. Next, we blocked endogenous DMT1 isoforms using ebselen, a potent pharmacological inhibitor of DMT1 iron transport. Ebselen pre-treatment blocked morphine’s ability to upregulate FHC protein, suggesting this pathway requires DMT1 iron transport from endolysosomes. This was further validated using viral-mediated genetic silencing of DMT1±IRE in cortical neurons, which also blocked FHC upregulation in the presence of morphine. Overall, our work demonstrates that the μ-opioid agonist morphine utilizes the endolysosomal iron transporter DMT1 to modulate neuronal cellular iron metabolism, upregulate FHC protein, and contribute to cognitive decline in neuroHIV.

**Graphical Abstract:**

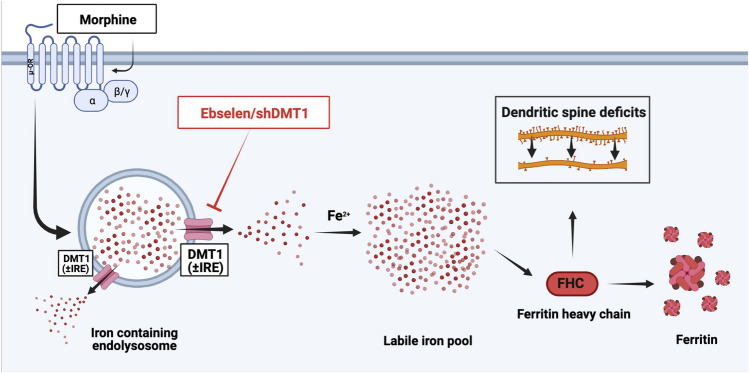

Morphine requires DMT1 to upregulate neuronal FHC. Cortical neurons treated with morphine release their endolysosomal iron stores to the cytoplasm and upregulate FHC, an iron storage protein associated with dendritic spine deficits and cognitive impairment in neuroHIV. This pathway requires the endolysosomal iron transporter DMT1, as pharmacological and genetic inhibitors of the transporter completely block morphine’s ability to upregulate FHC. Created with BioRender.com.

## Introduction

Several neurologic disorders present with dysregulated cellular iron metabolism in the central nervous system, which can drive neuroinflammatory processes and disease progression (Rouault 2013; Porras and Rouault 2022). Brain iron metabolism has notably been examined in the context of Alzheimer’s (Lane et al. 2018) and Parkinson’s (Mochizuki et al. 2020) diseases, and more recently in neuroHIV (Pitcher et al. 2014; Chang et al. 2015; Patton et al. 2017; Fennema-Notestine et al. 2020), a neurologic disorder associated with HIV infection of select brain cells. Although HIV infection can be well-controlled by antiretroviral therapies, people living with HIV often develop mild to moderate cognitive impairment that can disrupt their daily life (Saylor et al. 2016). Our group and others have reported that HIV infection and common comorbidities like opioid use are associated with dysregulated iron metabolism in the brain, which could be an important factor that leads to cognitive impairment in people with HIV (Pitcher et al. 2014; Nash et al. 2021).

Cellular iron levels are normally tightly controlled by an elegant iron regulatory system. The system senses labile (free) iron within the cell and responds by adjusting the translation of proteins that import, store, and export iron (Singh et al. 2014; Gao et al. 2019). Intriguingly, cortical neurons upregulate the iron storage protein ferritin heavy chain when exposed to µ-opioid agonists (Sengupta et al. 2009; Pitcher et al. 2014). This occurs through a µ-opioid receptor signaling pathway that increases endolysosome pH and causes these organelles to release their iron stores into the cytoplasm (Nash et al. 2019). The pathway can be blocked by chelating endolysosomal iron, suggesting that µ-opioids specifically regulate intracellular iron stores. Notably, endolysosome iron chelation also blocked morphine’s ability to reduce cortical dendritic spines (Nash et al. 2019), which are key mediators of learning and memory (Nimchinsky et al. 2002). This suggests morphine or other µ-opioid agonists could contribute to cognitive impairment in people with HIV by dysregulating neuronal iron storage or metabolism. However, it is still unclear how µ-opioid signaling encourages endolysosomes to release their iron stores in central nervous system neurons.

We started to answer this question by determining if morphine regulates the endolysosomal iron transporter divalent metal transporter 1 (DMT1). DMT1, also known as solute carrier family 11 member 2 (SLC11A2), natural resistance associated macrophage protein 2 (NRAMP2), and divalent cation transporter 1 (DCT-1), is a pH-sensitive divalent cation transporter expressed in most cells (Gunshin et al. 1997; Yanatori and Kishi 2019). There are four isoforms of DMT1, characterized by small changes of the 5’ (1A, 1B) and 3’ (+IRE, -IRE) ends of the transporter (Mackenzie et al. 2007). Only the 1B isoforms are expressed in neurons (Pelizzoni et al. 2012; Skjorringe et al. 2015), which can contain or lack an iron responsive element (IRE) in the 3’ untranslated region. In this study, we precisely characterized the expression and subcellular localization of DMT1 1B isoforms in primary rat cortical neurons and examined if morphine requires the transporter to increase ferritin heavy chain protein levels in these cells. We also rigorously tested the specificity of our experimental approaches using several DMT1 reporter constructs and inhibition or knockdown strategies. This study provides further evidence that µ-opioids regulate neuronal iron metabolism and presents a new and improved framework for future studies of DMT1 in brain cells.

## Methods

### Animals

This study used E17 Holtzman rat embryos to produce primary cultures of cortical neurons. We obtained embryos from time-pregnant dams (HsdHot:Holtzman SD) purchased from Envigo (order code: 003). One experiment compared primary cortical neurons from Holtzman and Sprague Dawley E17 embryos, obtained from Sprague Dawley time-pregnant dams (Hsd:Sprague Dawley SD), also purchased from Envigo (order code: 002). Male F344 rats (F344/NHsd) purchased from Envigo (Order code: 010) were used to collect frontal cortex homogenates at 6 months old. All animals were kept in Association for Assessment and Accreditation of Laboratory Animal Care-accredited university facilities in accordance with the National Institutes of Health guidelines and institutional approval by the Institutional Animal Care and Use Committee (protocol numbers LA-21-613 – renewed 4/1/22, LA-21-614 – renewed 4/1/22, PHS Animal Welfare Assurance D16-00138). Dams were housed individually, and male F344 rats were pair housed. Both sets of rats had uninterrupted access to food and water prior to sacrifice by CO_2_ inhalation.

### Primary Rat Neuronal Cultures

We prepared cultures of primary rat cortical neurons as described previously (Sengupta et al. 2009) and originally in (Brewer et al. 1993). Briefly, cortical tissue was dissected from mixed male and female E17 Holtzman rat embryos, trypsinized (ThermoFisher 15090046, 0.25% final concentration) for 15 min in a 37 °C water bath, and then exposed to DNAse (Sigma-Aldrich D5025, 60 µg/mL) and triturated to form a single cell suspension. The cell suspension was mixed with an equal amount of Neurobasal media (ThermoFisher 21103049) containing 2% donor equine serum (Hyclone SH3007403HI), 2% B-27 supplement 50X (ThermoFisher 17504044), 0.5 mM GlutaMAX (ThermoFisher 35050061), and 25 µM L-glutamic acid (Tocris 0218). Cells were counted with a hemocytometer and plated in 60 mm culture dishes (VWR 25382-188, 1 million cells) or 15 mm glass coverslips (Carolina 633031, 35,000 cells), both pre-coated with poly-L-lysine (Sigma-Aldrich P1274). After 3–4 h, culture media was changed to the same Neurobasal medium formula but without donor equine serum. On the second day in vitro, cytosine β-D-arabinofuranoside (Sigma-Aldrich C6645) was added to cultures (1 µM per dish, 0.5 µM per coverslip) to prevent glial proliferation, and culture medium was replaced every 4 days thereafter with Neurobasal medium containing 2% B-27 and 0.5 mM GlutaMAX. Neurons were used for experiments at 12 days in vitro.

### Cell Cultures and Transfections

Human embryonic kidney 293 T cells (HEK-293) were purchased from ATCC (CRL-3216), and Lenti-X 293 T cells were purchased from Takara (632,180). HEK-293 were used for immunofluorescence, western blot, and qPCR experiments. Lenti-X 293 T cells were used for viral production, as this subclone is highly transfectable and supports high levels of viral expression. The cells were stored, thawed, and prepared for cell culture according to the manufacturer’s instructions. Cells were cultured in DMEM containing 10% fetal bovine serum (Hyclone SH3007003HI) and 50 µg/mL gentamicin (Life Technologies 15750–060). Upon reaching confluency, cultures were split and replated at a 1:10 ratio using trypsin–EDTA 1X (Gibco 25–200-056) and fresh culture medium. Cells were used for experiments and viral production for up to 10 passages. For transfections, cells were seeded on 12 well plates the day before treatment at 160,000 cells/well. Lipofectamine 2000 (ThermoFisher 11668019) – DNA complexes (2 μl of Lipofectamine 2000 per transfection) were prepared in OptiMEM (Gibco 31-985-062) according to manufacturer instructions, incubated for 5 min at room temperature in the dark, and transferred dropwise on coverslips. After a 2.5-h incubation at 37 °C, transfection medium was removed and replaced with fresh medium.

### Reagents

Morphine sulfate salt pentahydrate (Sigma-Aldrich M8777) was dissolved in ultrapure water, passed through 0.2 µM syringe filters into sterile Eppendorf tubes, and frozen at –20 °C in the dark until use. Ebselen (Tocris 5245) and ferric ammonium citrate (Sigma-Aldrich F5879) were prepared fresh for each experiment according to the manufacturer’s instructions.

### Construction of Plasmids and Viral Particles

We obtained a plasmid containing the rat DMT1 sequence (NCBI Ref seq: AF029757.1) fused to gfp from SinoBiological (RG80867-ACG). The sequence encoding the fusion protein was amplified and inserted in the 3^rd^ generation lentiviral transfer vector pUltraHot, a gift from Malcolm Moore (Addgene plasmid # 24130; http://n2t.net/addgene:24130; RRID:Addgene_24130), between sites AgeI-EcoRI, to generate UHDMT1(-IRE)g. We followed the same strategy to generate a DMT1-IRE transfer vector without a gfp tag by introducing the DMT1-IRE coding sequence in pUltraHot and named the plasmid UHDMT1(-IRE). To generate the transfer vector expressing DMT1(+IRE) with C-terminal fluorescent tag, the DMT1 and mCherry fluorescent protein sequences were amplified with primers sharing a partially overlapping sequence encoding the VSISKVLLSEDTSGGNTK motif in DMT1(+IRE). The two PCR products were then joined in the lentiviral transfer vector pUltraHot and named UHDMT1(+IRE)mC. To obtain UHDMT1(+IRE), the mCherry sequence was removed as described above for UHDMT1(-IRE). Plasmids were assembled using the Gibson assembly strategy (NEBuilder HiFi DNA Assembly Cloning Kit, NEB #E5520, New England Biolabs). Fragments were amplified with Q5 High-Fidelity 2X Master Mix (NEB #M0492S, New England Biolabs). The pLKO.1 vector used to introduce shRNAs targeting DMT1 was a gift from Bob Weinberg (Addgene plasmid # 8453; http://n2t.net/addgene:8453; RRID:Addgene_8453). Targeting sequences were selected using the shRNA target design tool from VectorBuilder (https://en.vectorbuilder.com/tool/shrna-target-design.html). The four sequences (shRNA-1: TGGAGCAGTGGCTGGATTTAA; shRNA-3: GGCAATCATTGGTTCTGATAT; shRNA-4: ACTATCATGGCCCTCACATTT; shRNA-5: GGAAGTTCGAGAAGCCAATAA) and the corresponding (-) strands were synthesized with protruding overhangs generating AgeI-EcoRI sticky ends. Paired oligos were annealed and cloned in the AgeI-EcoRI digested pLKO.1 vector, using the Takara ligation kit version 2.1 (#6022). Restriction enzymes were purchased from New England Biolabs. Oligos were synthesized by Integrated DNA Technologies. HEK-293 cells (Lenti-X^™^ 293 T Cell Line, #632180, Takara) grown on 10 cm dishes were co-transfected with the appropriate transfer vectors and packaging plasmids pCMVR8.74 (Addgene plasmid # 22036; http://n2t.net/addgene:22036; RRID:Addgene_22036) and pMD2.G (Addgene plasmid # 12259; http://n2t.net/addgene:12259; RRID:Addgene_12259), both gifts from Didier Trono. Viral particles released in the media of transfected cells were concentrated through ultracentrifugation and stored at -80 °C.

### Western Blots

Western blots were performed as previously described (Nash et al. 2019). For total protein extracts, cultured cells were lysed using a triple detergent buffer with protease inhibitors (ThermoFisher 1861278), and phosphatase inhibitors (Millipore 524625). Lysates were centrifuged at 14,000 rpm at 4 °C for 10 min, and resulting supernatants were transferred to new Eppendorf tubes and supplemented with a 50% glycerol solution (Sigma-Aldrich G6279, 10µL per 50µL protein lysate) prior to storage at -20 °C.

For cytosolic and nuclear extracts, plasma membranes were ruptured using a hypotonic buffer solution with 1 mM 4-(2-Aminoethyl)benzenesulfonyl fluoride hydrochloride (AEBSF) (Sigma-Aldrich A8456), 5 µg/ml aprotinin (Sigma-Aldrich A4529), 5 µg/ml leupeptin (Sigma-Aldrich L2884), and 5 µg/ml pepstatin A (Sigma-Aldrich P5318). Then IGEPAL CA-630 (Sigma-Aldrich I8896 0.05%) was added to each lysate, followed by centrifugation at 14,000 rpm for 2 min at 4 °C to pellet the nuclear fraction. The supernatant containing the cytosolic fraction was stored in a new Eppendorf tube, supplemented with a 50% glycerol solution (Sigma-Aldrich G6279, 10µL per 50µL of extract), and frozen at -80 °C. The nuclear fraction in the pellet was lysed using the triple detergent buffer described above, on ice with vortexing every 10 min for 45 min. The nuclear lysate was then centrifuged at 14,000 rpm for 30 min at 4 °C and the supernatant was transferred to a new tube and supplemented with glycerol to be stored at -80 °C.

Protein lysate concentration was estimated using a Pierce bicinchoninic acid assay kit (ThermoFisher 23225) according to the manufacturer’s instructions. Each Western blot sample contained 30 – 40 µg of protein. Samples to be probed for DMT1 isoforms were not boiled prior to gel electrophoresis, whereas all other samples were boiled. Boiled and unboiled sample preparations were run on different gels, which better identified target proteins. Protein samples were run on a 12.5% bis-acrylamide gel (to probe ferritin heavy chain) or an 8% tris-tricine gel (for all other targets) followed by transfer onto a polyvinylidene fluoride (PVDF) membrane (Millipore IPVH00010) for immunoblotting. PVDF membranes were blocked with NAP-Blocker (VWR 82022-626) in PBS prior to addition of primary and secondary antibodies (Table 1). Membranes were exposed to the Pierce ECL Western Blotting Substrate (ThermoFisher 32106) for densitometry using an Alpha Innotech Fluorchem IS-8900 gel imaging system. Band densities were quantified using Fiji version 2.5.0 (RRID:SCR_002285). Membranes to be probed with additional antibodies were stripped using a Restore Western Blot Stripping Buffer (ThermoFisher 21059) according to the manufacturer’s instructions.

**Table 1:** Antibodies used for Western blots

**Primary antibody**	**Company and catalog number**	**RRID**	**Dilutions**
DMT1+IRE	Alpha Diagnostic NRAMP21-A	RRID:AB_1620356	1:300
DMT1-IRE	Alpha Diagnostic NRAMP23-A	RRID:AB_1620359	1:300
GFP (B-2)	Santa Cruz Biotechnology sc-9996	RRID:AB_627695	1:1000
mCherry	Abcam ab183628	RRID:AB_2650480	1:3000
Ferritin heavy chain (FHC)	Cell Signaling Technology 3998S	RRID:AB_1903974	1:1000
Actin	Sigma-Aldrich A2066	RRID:AB_476693	1:5000
Histone H3	Cell Signaling Technology 9715S	RRID:AB_331563	1:1000
GAPDH	Cell Signaling Technology 5174S	RRID:AB_10622025	1:1000

**Secondary antibody**	**Company and catalog number**	**RRID**	**Dilutions**
HRP-conjugated goat anti-rabbit IgG	Invitrogen 31460	RRID:AB_228341	1:50000
HRP-conjugated goat anti-mouse IgG	Pierce 1858413 (Invitrogen 32430)	RRID:AB_1185566	1:2000

### Immunocytochemistry

Immunocytochemistry was performed as described previously (Festa et al. 2020). Cells on coverslips were fixed using a 4% ice-cold paraformaldehyde (PFA) (Electron Microscopy Sciences 19208) solution for 20 min. The cells were then permeabilized with 0.1% Triton-X 100 (Sigma T9284) in PBS, blocked with 5% normal goat serum (Jackson ImmunoResearch 005-000-121), and then incubated with a primary antibody (Table 2) in blocking solution overnight at 4 °C. The next day, coverslips were washed with PBS prior to addition of the secondary antibody (Table 2) in blocking solution for 30 min at room temperature in the dark. This process was repeated for additional primary antibodies, as needed. Next, coverslips were counterstained with Hoechst (ThermoFisher H3570, 1:10,000 in PBS), washed in distilled deionized Millipore water, and mounted on microscope slides using ProLong Gold Antifade Reagent (ThermoFisher P36930). Mounted coverslips were sealed with nail polish and stored at -20 °C in the dark until imaging with an Olympus Fluoview FV3000 confocal laser scanning microscope (RRID:SCR_017015).

**Table 2:** Antibodies used for immunocytochemistry

**Primary antibody**	**Company and catalog number**	**RRID**	**Dilutions**
DMT1+IRE	Alpha Diagnostic NRAMP21-A	RRID:AB_1620356	1:200
DMT1-IRE	Alpha Diagnostic NRAMP23-A	RRID:AB_1620359	1:200
LAMP1	Enzo Life Sciences ADI-VAM-EN001-D	RRID:AB_2038958	1:100
MAP-2 (mouse)	Abcam ab11267	RRID:AB_297885	1:500
MAP-2 (chicken)	Abcam ab92434	RRID:AB_2138147	1:500

**Secondary antibody**	**Company and catalog number**	**RRID**	**Dilutions**
Alexa Fluor 647 goat anti-mouse IgG	Invitrogen A-21235	RRID:AB_2535804	1:500
Alexa Fluor 488 goat anti-mouse IgG	Invitrogen A-11001	RRID:AB_2534069	1:500
Alexa Fluor 568 goat anti-mouse IgG	Invitrogen A-11004	RRID:AB_2534072	1:500
Alexa Fluor 488 goat anti-rabbit IgG	Invitrogen A-11008	RRID:AB_143165	1:500
Alexa Fluor 568 goat anti-rabbit IgG	Invitrogen A-11011	RRID:AB_143157	1:500
Alexa Fluor 647 goat anti-chicken IgG	Invitrogen A-32933	RRID:AB_2762845	1:5000

### Quantitative Real-Time Polymerase Chain Reaction

RNA was collected from cultured cells using a RNeasy Plus Mini Kit (Qiagen 74134) following the manufacturer’s instructions. The purity of the extraction and the amount of RNA collected were analyzed using a NanoDrop ND-100 spectrophotometer. Cellular RNA (250 ng) was used to produce cDNA with Oligo(dT)20 Primer (ThermoFisher 18418020), a Deoxynucleotide (dNTP) Solution Mix (New England BioLabs N0447S) and SuperScript III Reverse Transcriptase (Invitrogen 18–080-044) according to the manufacturer’s instructions. cDNA was stored at -20 °C until use. For real-time quantitative PCR studies, TaqMan Fast Advanced Master Mix (Applied Biosystems 4444556) was prepared according to the manufacturer’s instructions. Primer sequences included rat DMT1 TaqMan gene express array (ThermoFisher 4351370, assay id: Rn01553109_m1), rat GAPDH TaqMan gene express array (ThermoFisher 4351370, assay id: Rn01749022_g1) and human GAPDH TaqMan gene express array (ThermoFisher 4331182, assay id: Hs02786624_g1). Master mix solutions containing primers and cDNA were added to a MicroAmp Fast 96-well reaction plate 0.1 mL (Applied Biosystems 4346907), covered with adhesive film (Applied Biosystems 4306311), and placed in a QuantStudio 7 Flex Real-Time PCR System (Applied Biosystems 4485701) for quantitative PCR. Samples were run in triplicate (3 technical replicates) and data was quantified using the delta-delta CT method.

### Experimental Design and Statistical Analysis

Animals used for ex-vivo and neuronal culture studies were randomly assigned to groups. Biological replicates use neurons derived from different litters of E17 rats, and technical replicates use neurons collected from the same litter. All reported experiments have at least three biological replicates, and the exact N of biological replicates is reported in the figure legends. Quantitative PCR analyses include three technical replicates per condition, which were averaged to one value per condition.

We used Graphpad Prism version 9.1.1 (RRID:SCR_002798) to generate graphs and analyze the data obtained in this study. All data are presented as mean ± standard error of the mean. Normally distributed datasets with two independent groups were analyzed using a two-tailed Student’s t-test, while datasets with more than two independent groups were analyzed using either one-way ANOVA and Tukey’s or Dunnett’s post hoc or two-way ANOVA and Sidak post hoc. Immunofluorescence colocalization data was analyzed using the Just Another Colocalization Plugin (JACoP) in Fiji version 2.5.0 (RRID:SCR_002285) to generate Pearson’s correlation coefficients. Figure legends report the statistical tests used for each experiment. Data analyses reached statistical significance at p < 0.05, and p values are reported in figure legends. All raw data is available upon request.

## Results

### Validation of DMT1 Antibodies

**Fig. 1:**
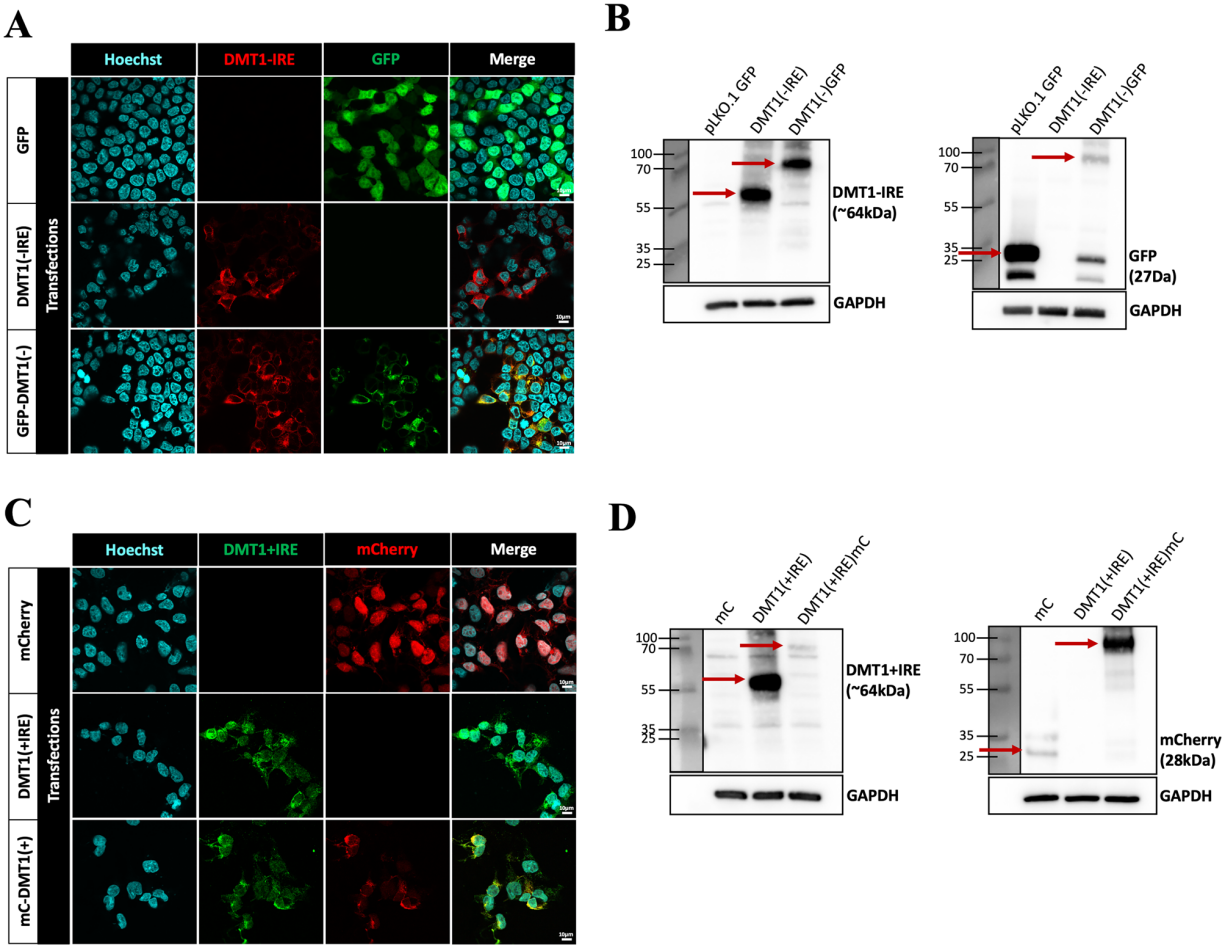
Validation of DMT1 antibodies. **A** Confocal micrographs showing expression of DMT1-IRE in HEK-293 cells transfected with either GFP (top row), DMT1-IRE (middle row) or a GFP-tagged DMT1-IRE (bottom row). Hoechst counterstain shows cell nuclei. N = 3. Scale bar = 10 µm. **B** Western blots showing DMT1-IRE expression in protein lysates of transfected HEK-293 cells. The DMT1-IRE antibody (left blot) identified DMT1-IRE alone (64 kDa) and GFP-DMT1-IRE (92 kDa), and the GFP antibody (right blot) identified GFP alone (27 kDa) and GFP-DMT1-IRE (91 kDa). GAPDH antibody shows protein loading. N = 3. **C** Confocal micrographs showing expression of DMT1+IRE in HEK-293 cells transfected with either mCherry (top row), DMT1+IRE (middle row), or a mCherry-tagged DMT1+IRE (bottom row). N = 3. Scale bar = 10 µm. **D** Western blots showing DMT1+IRE expression in protein lysates of transfected HEK-293 cells. The DMT1+IRE antibody (left blot) identified DMT1+IRE alone (64 kDa) and mCherry-DMT1+IRE (92 kDa), and the mCherry antibody (right blot) identified mCherry alone (28 kDa) and mCherry-DMT1+IRE (92 kDa). GAPDH antibody shows protein loading. N = 4

We began by validating antibodies that target either the +IRE or -IRE isoform of rat DMT1 using an HEK-293 cell-based expression system. We transfected HEK-293 cells with constructs that expressed either a rat DMT1 isoform alone, an isoform tagged with a fluorescent protein, or the fluorescent protein alone, and we used these cells to examine DMT1 ± IRE expression in Western blotting and immunocytochemistry. In cells transfected with rat DMT1-IRE (±GFP), we observed strong cytoplasmic staining with the DMT1-IRE antibody that was absent from cells transfected with GFP alone. DMT1-IRE staining also overlapped the GFP signal in cells expressing GFP-DMT1-IRE (Fig. 1A). In Western blot studies, the DMT1-IRE and GFP antibodies detected GFP-tagged DMT1-IRE at 92 kDa as well as their targeted proteins alone (DMT1-IRE at 64 kDa and GFP at 27 kDa). The antibodies showed little to no non-specific signal when their target protein was absent from a cell lysate (Fig. 1B).

In cells transfected with rat DMT1+IRE (±mCherry), we observed ample intracellular staining with the DMT1+IRE antibody, which was absent in cells transfected with mCherry alone. DMT1+IRE staining also strongly overlapped the mCherry signal in cells expressing mCherry-DMT1+IRE (Fig. 1C). In Western blot studies, the DMT1+IRE and mCherry antibodies detected mCherry-tagged DMT1+IRE at 92 kDa as well as their targeted proteins alone (mCherry at 28 kDa, and DMT1+IRE at 64 kDa). As before, both antibodies showed little to no non-specific signal when their targeted protein was absent from a cell lysate (Fig. 1D). Therefore, DMT1 isoform specific antibodies bind their targeted proteins as well as GFP/mCherry tagged versions. These studies also confirmed the molecular weight of rat DMT-1 isoforms on the western blot and validated the DMT1 constructs for use as positive controls in experiments that examine endogenous expression of rat DMT1 isoforms.

### Endogenous and Exogenous Expression of DMT1 in Rat Cortical Neurons

**Fig. 2:**
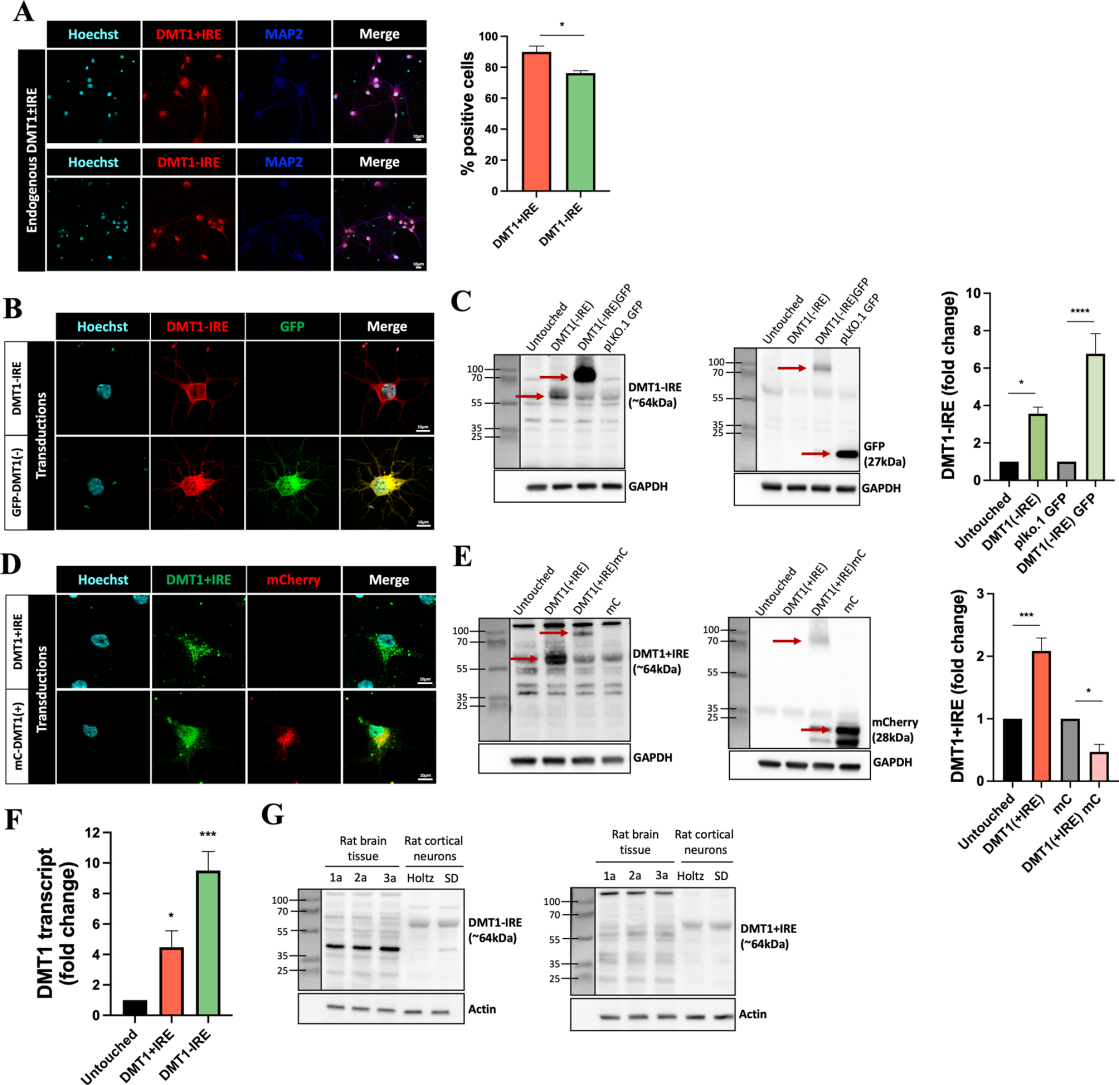
Endogenous and exogenous expression of DMT1 isoforms in rat cortical neurons. **A** Confocal micrographs of primary neurons stained for endogenous DMT1-IRE or DMT1+IRE protein, MAP2 (neuronal marker) and Hoechst (cell nuclei). Scale bar = 10 µm. Quantification of positive cells [%] for DMT1 isoforms is shown in the adjacent graph. For each experiment, positive cells were counted from 20 micrographs and averaged into a single data point. N = 3. **B** Confocal micrographs showing DMT1-IRE staining in primary neurons transduced with DMT1-IRE constructs. Hoechst counterstain shows cell nuclei. N = 3. Scale bar = 10 µm. **C** Western blots showing DMT1-IRE expression in transduced primary neurons. The DMT1-IRE and GFP antibodies identified their target proteins and a high molecular weight band corresponding to the GFP-tagged DMT1-IRE fusion protein. GAPDH antibody shows protein loading. N = 4, one-way ANOVA with Dunnett’s post hoc, *p = 0.00356, ****p < 0.0001. **D** Confocal micrographs showing DMT1+IRE staining in primary neurons transduced with DMT1+IRE constructs. Hoechst counterstain shows cell nuclei. N = 3. Scale bar = 10 µm. **E** Western blots showing DMT1+IRE expression in transduced primary neurons. Like in C, the DMT1+IRE and mCherry antibodies identified their target proteins as well as the higher molecular weight band corresponding to the fusion protein. GAPDH antibody shows protein loading. N = 4, one-way ANOVA with Dunnett’s post hoc, ***p = 0.0001, *p = 0.0228. **F** Quantitative PCR of DMT1±IRE expression in transduced primary neurons. N = 3, one-way ANOVA with Dunnett’s post hoc, *p = 0.0438, ***p = 0.0002. **G** Western blots showing endogenous DMT1±IRE expression from frontal cortex lysates of adult rats. Primary cortical neurons from two different rat strains are included for comparison. Actin antibody shows protein loading. N = 3 adult rats, one per lane

We next used the validated antibodies to examine endogenous expression of DMT1 isoforms in primary rat cortical neurons. Cortical neuron cultures showed robust DMT1-IRE and DMT1+IRE staining that colocalized with the neuronal marker microtubule-associated protein 2 (MAP2) in the neuronal soma. Indeed, most cultured neurons were positive for DMT1-IRE or DMT1+IRE staining, as determined by quantitative analysis of the immunofluorescence data (Fig. 2A).

We studied endogenous and exogenous expression of DMT1 isoforms in this neuronal culture system. Primary rat cortical neurons were either untouched or transduced with a DMT1 expression construct or empty vector from Fig. 1, and we examined DMT1 isoform expression in these conditions using similar approaches. As expected, neurons transduced with DMT1-IRE (±GFP) showed robust staining with the DMT1-IRE antibody that colocalized with the GFP signal when present (Fig. 2B). In Western blot studies, the DMT1-IRE antibody identified endogenous and exogenous DMT1-IRE at the same molecular weight in addition to the heavier GFP-tagged version. Probing the blot with a GFP antibody identified the tagged DMT1-IRE as well as GFP alone in neurons transduced with these proteins (Fig. 2C). Our companion studies examining DMT1+IRE produced similar findings. Neurons transduced with DMT1+IRE (±mCherry) showed robust staining with the DMT1+IRE antibody that colocalized with the mCherry signal when present (Fig. 2D). In Western blot studies, the DMT1+IRE antibody identified endogenous and exogenous DMT1+IRE at the same molecular weight in addition to the heavier mCherry-tagged protein. As before, probing the blot with a mCherry antibody identified the tagged DMT1+IRE and mCherry alone in neurons expressing these proteins (Fig. 2E). These studies demonstrate DMT1 antibodies label endogenous and exogenous DMT1 isoforms in immunostaining approaches and at the expected molecular weight in the Western blot.

We also examined DMT1±IRE mRNA levels in untouched and transduced cortical neurons as an additional control for our studies at the protein level. As expected, transduced neurons showed significantly higher DMT1±IRE mRNA levels than untouched neurons (Fig. 2F). Interestingly, neurons transduced with the DMT1-IRE construct expressed about double the amount of transcript as their DMT1+IRE transduced counterparts, suggesting that cortical neurons may differentially regulate transcription of each DMT1 isoform in basal iron conditions.

Finally, we examined the protein level of DMT1 isoforms in frontal cortex lysates from adult F344 rats. These samples showed several diffuse bands around the molecular weight that we observe DMT1 in primary cortical neurons from Holtzman and Sprague Dawley rats (Fig. 2G), which could be due to less overall DMT1 in the sample and/or posttranslational modifications of these proteins in vivo (Gruenheid et al. 1999). Therefore, the DMT1±IRE antibodies can detect DMT1 isoforms in several different sample preparations and experimental approaches.

### DMT1 Isoforms Subcellular Localization

**Fig. 3:**
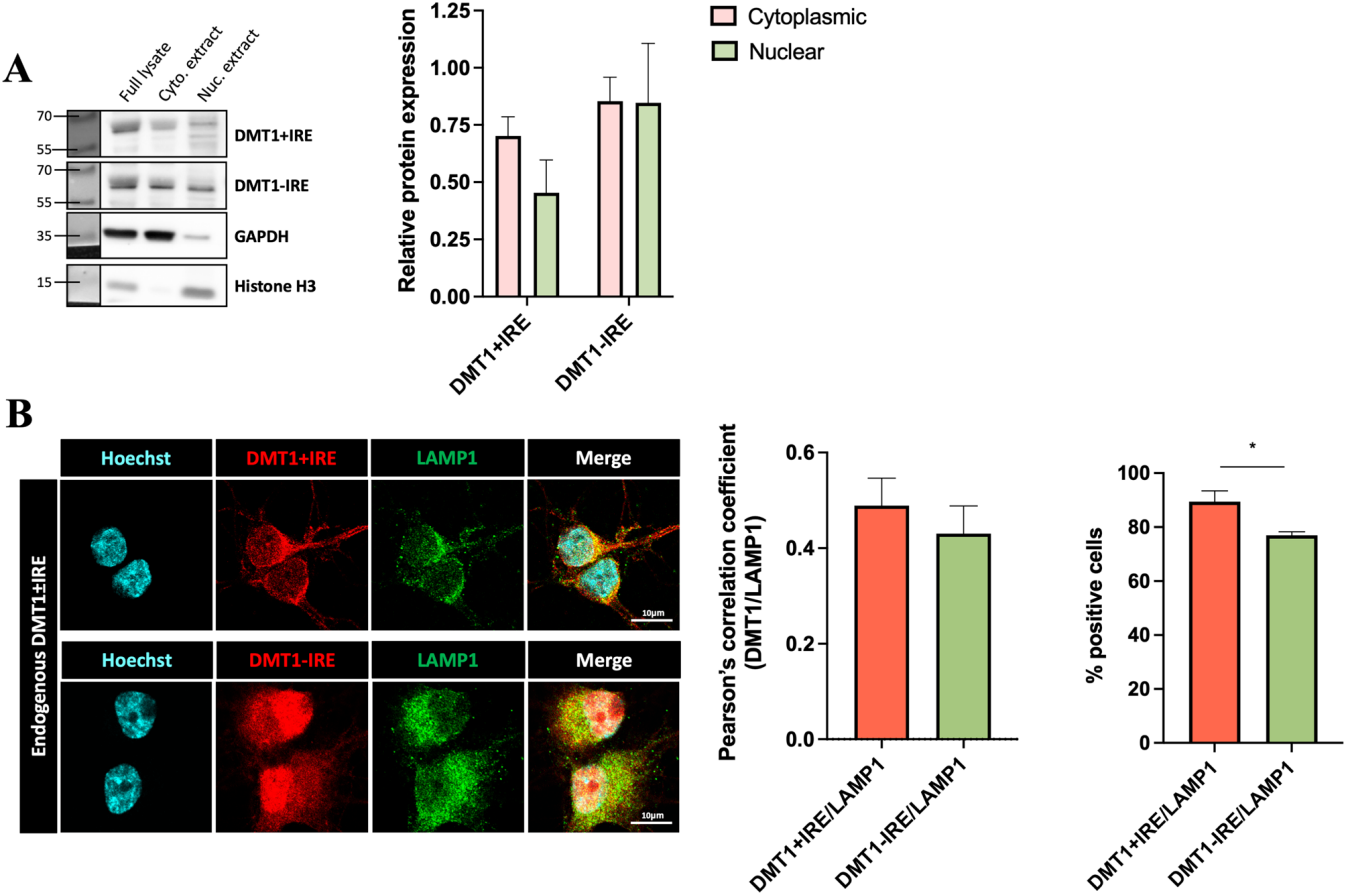
Subcellular localization of endogenous DMT1 isoforms. **A** Western blots showing DMT1±IRE protein levels in cytoplasmic and nuclear extracts of primary neurons. Extraction controls include the cytoplasmic protein GAPDH and the nuclear protein histone H3. Relative expression of DMT1 isoforms is quantified in the adjacent graph. N = 5, two-way ANOVA with Sidak’s post hoc Cytoplasmic (+IRE) vs Cytoplasmic (-IRE) p = 0.5072, Nuclear (+IRE) vs Nuclear (-IRE) p = 0.9994. **B** Confocal micrographs of primary neurons stained for endogenous DMT1 isoforms, the endolysosomal marker LAMP1, and Hoechst stain for cell nuclei. Scale bar = 10 µm. DMT1 isoforms and LAMP1 colocalization quantified in the adjacent graph. N = 3, unpaired Student’s t-test, p = 0.5121, *p = 0.0414

Our next goal was to confirm DMT1 expression on endolysosomes of primary rat cortical neurons and examine the subcellular localization of DMT1 isoforms in these cells more broadly. To start, we examined DMT1 isoforms in cytoplasmic and nuclear extracts of primary neurons via Western blot (Fig. 3A). DMT1±IRE isoforms were expressed in the cytoplasmic extracts, which places them in the same general compartment as neuronal endolysosomes. Both isoforms appear to be present in the nuclear extracts as well. Additional DMT1+IRE bands from these extracts were located below the expected molecular weight, which could be due to lack of posttranslational modifications or nonspecific antibody binding. The extractions were very pure, with minimal cross-contamination between the cytoplasmic marker glyceraldehyde-3-phosphate dehydrogenase (GAPDH) and the nuclear marker histone H3.

Next, we stained primary neurons for a single isoform of DMT1 and the endolysosomal marker lysosomal-associated membrane protein 1 (LAMP1), followed by a counterstain for cell nuclei (Fig. 3B). As shown in the figure, both DMT1 isoforms colocalized with LAMP1 staining, albeit to a different extent, suggesting that they are expressed on neuronal endolysosomes. This is in line with previous reports (Tabuchi et al. 2000; Pelizzoni et al. 2012). We used these immunofluorescence data to quantify the signal colocalization as well as the percent of neurons with colocalized signals (Fig. 3B). We observed relatively high, but not total colocalization of DMT1±IRE and LAMP1 throughout these neurons, and most neurons were positive for colocalized DMT1±IRE and LAMP1 signals. Therefore, both DMT1 isoforms are well-positioned to transport iron from endolysosomal stores to the cytoplasm in primary cortical neurons.

### Pharmacological Inhibition of DMT1

**Fig. 4:**
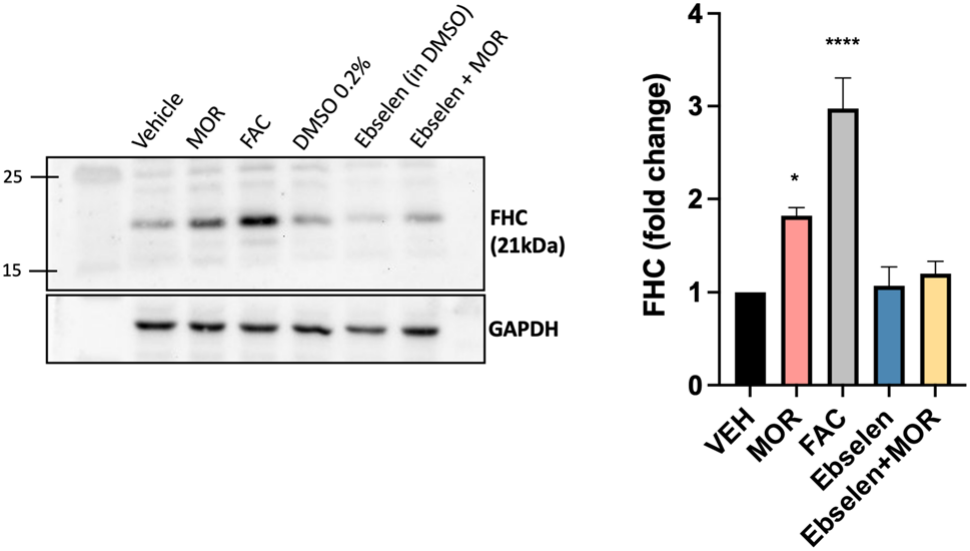
Pharmacological inhibition of DMT1. Western blot showing ferritin heavy chain (FHC) protein levels in primary neurons treated with either morphine (1 µM, 24 h), ebselen (10 µM, 24.5 h), or pre-treated with ebselen for 0.5 h followed by morphine treatment as above. Ferric ammonium citrate (FAC) (25 µM, 24 h) used as a positive control to increase FHC protein level. Vehicle controls include ddH2O for morphine and 0.2% DMSO for ebselen. FHC expression quantified in adjacent graph. GAPDH antibody shows protein loading. N = 5, one-way ANOVA with Dunnett’s post hoc, *p = 0.0315, **** p < 0.001

We previously showed that long-term morphine exposure upregulates ferritin heavy chain protein in cortical neurons (Sengupta et al. 2009; Pitcher et al. 2014) via a process that releases endolysosomal iron stores into the cytoplasm (Nash et al. 2019). As DMT1 often facilitates endolysosomal iron release to the cytoplasm and other organelles (Shawki et al. 2012), we asked if a pharmacological inhibitor of DMT1 could block morphine’s ability to upregulate ferritin heavy chain. To this end, we used ebselen - a potent inhibitor of DMT1 that was originally identified in a high-throughput screen (Wetli et al. 2006) and has since been used in several studies examining neurons (Howitt et al. 2009; Pelizzoni et al. 2012; White et al. 2016; Liu et al. 2018). We pre-treated primary neurons with 10 µM ebselen for 30 m, then added 1 µM morphine to the ebselen-containing media and collected cell lysates 24 h later. Additional controls included vehicle groups for both morphine (distilled deionized H_2_O) and ebselen (0.2% DMSO), as well as a positive control of 25 µM ferric ammonium citrate (FAC) since FHC levels are directly regulated by labile iron (Torti and Torti 2002). Morphine alone increased FHC protein levels, and this was completely blocked by pre-treatment with ebselen, suggesting morphine requires DMT1 to upregulate FHC (Fig. 4). As expected, primary cortical neurons upregulated FHC in response to ferric ammonium citrate, while the vehicles for morphine and ebselen had no effect.

### Validation of DMT1 Knockdown

**Fig. 5:**
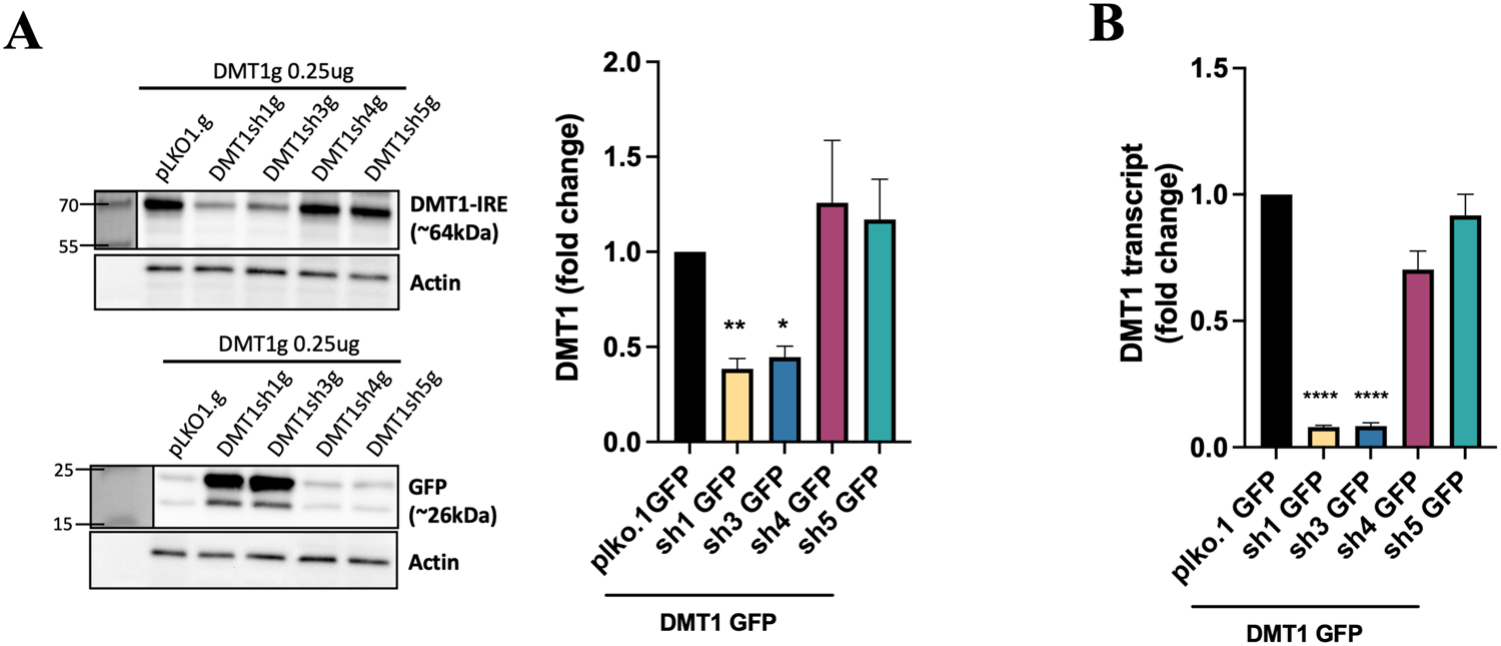
Validation of DMT1 knockdown. **A** Western blots showing protein levels of DMT1-IRE and GFP in HEK-293 cells co-transfected with a rat DMT1-IRE construct and one of several GFP-tagged DMT1-shRNA constructs. GFP-tagged plko1.g used as an empty vector control for the shRNA constructs. Actin shows protein loading. DMT1-IRE knockdown quantified in the adjacent graph. N = 3, one-way ANOVA with Dunnett’s post hoc, **p = 0.058, *p = 0.113 **B** Quantitative PCR analysis of DMT1-IRE expression in HEK-293 cells transfected with the same constructs as in A. N = 3, one-way ANOVA with Dunnett’s post hoc, **** p < 0.001

Ebselen is a highly effective inhibitor of DMT1, but it is relatively non-specific and could alter FHC levels via off-target anti-inflammatory effects (Santi et al. 2021). Therefore, we developed a strategy to knock down total DMT1±IRE using GFP-tagged DMT1 shRNA constructs that target outside the C-terminal ±IRE regions. We tested these constructs in HEK-293 cells transfected with a rat DMT1-IRE expression construct from Fig. 1 to determine the most effective shRNA construct. Of the four constructs tested, only sh1g and sh3g successfully knocked down DMT1 and strongly expressed GFP in the co-transfected cells (Fig. 5A). These results were mirrored in quantitative PCR analyses, where only sh1g and sh3g sharply decreased expression of DMT1 transcripts in the co-transfected cells (Fig. 5B).

**Fig. 6:**
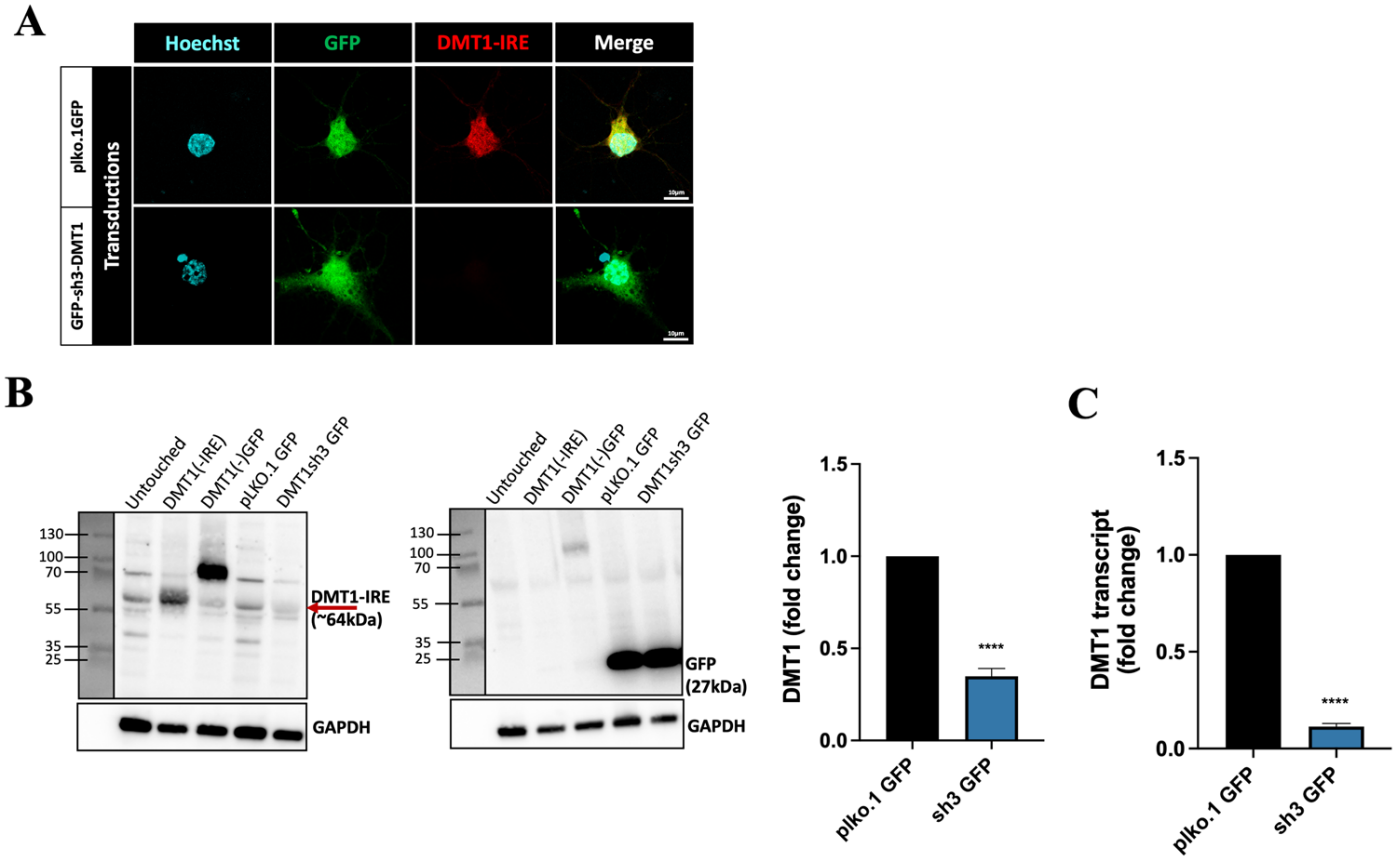
Knockdown of endogenous DMT1±IRE in primary neurons. **A** Confocal micrographs showing DMT1-IRE staining in primary neurons transduced with knockdown construct (GFP-sh3DMT) targeting DMT1±IRE and empty vector (GFP-plko.1). N = 3. Scale bar = 10 µm. **B** Western blots showing DMT1-IRE and GFP protein levels in primary neurons transduced with DMT1(-IRE) overexpression, GFP-DMT1(-IRE) overexpression, empty vector (GFP-plko.1) and knockdown construct (GFP-sh3DMT) targeting DMT1 ± IRE. GAPDH shows protein loading. N = 5, unpaired Student’s t-test, **** p < 0.001. **C** Quantitative PCR showing DMT1±IRE gene expression in primary neurons transduced with the same constructs as in A and B. N = 3, unpaired Student’s t-test, **** p < 0.001

We moved forward with sh3g for follow-up studies in primary neurons. As an initial test, we examined DMT1-IRE staining in neurons transduced with sh3g and the empty vector (Fig. 6A). Neurons transduced with empty vector showed staining with the DMT1-IRE antibody, but this staining was completely absent in neurons transduced with sh3g, which we identified using the GFP signal. Next, we quantified the sh3g-mediated knockdown in primary neurons using Western blotting and PCR. At the protein level, cultures transduced with sh3g had significantly less DMT1-IRE than those transduced with the control empty vector or exogenous DMT1-IRE (Fig. 6B). These results were mirrored at the mRNA level, where sh3g transduced cultures showed a 90% reduction of total DMT1±IRE transcripts compared to those transduced with the control empty vector (Fig. 6C). These results show that sh3g is an effective tool to knock down endogenous and exogenous DMT1±IRE in primary cortical neurons.

### Morphine Requires DMT1 to Upregulate Ferritin Heavy Chain in Cortical Neurons

**Fig. 7:**
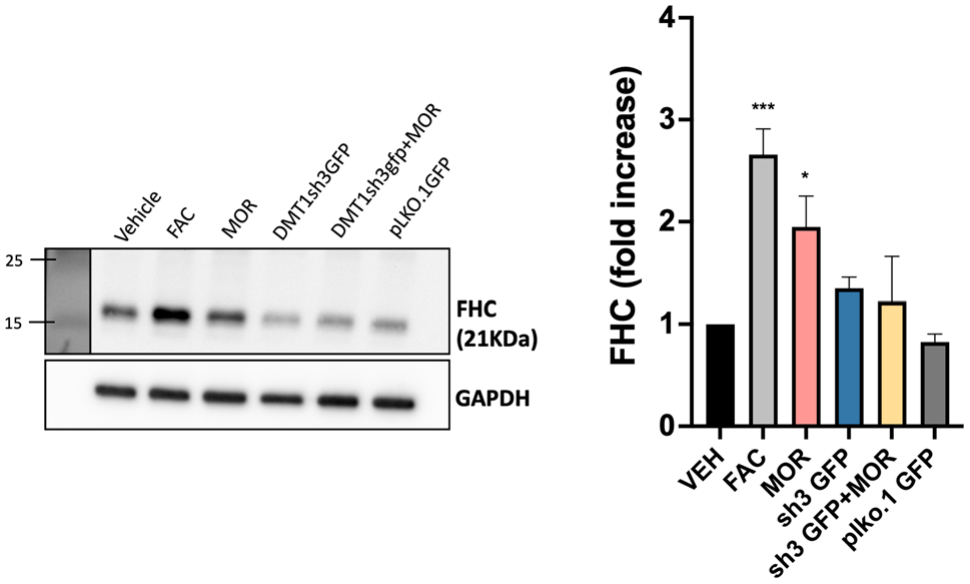
Morphine requires DMT1 to upregulate FHC in cortical neurons. Western blots showing FHC from primary neurons. Positive control includes ferric ammonium citrate (FAC), which upregulates FHC. The sh3g construct is controlled by the empty vector plko1g group. GAPDH used as a loading control. FHC expression quantified in adjacent graph. N = 5, one-way ANOVA with Dunnett’s post hoc, ***p = 0.0003, * p = 0.0465

Finally, we examined if morphine could still upregulate FHC protein in primary neurons transduced with the sh3g construct. At 6 days in vitro, we transduced cultures with constructs containing either sh3g or the control empty vector pLKO.1 g, which allowed the constructs ample time to be expressed before treatments. At 11 days in vitro, we treated the cultures with either 1 µM morphine or 25 µM FAC as a positive control to upregulate FHC, and we lysed the cultures 24 h later. In non transduced control cultures, morphine and FAC alone upregulated FHC protein levels as expected. However, morphine failed to upregulate FHC in sh3g transduced cultures, demonstrating that DMT1 is required for this process (Fig. 7). Together with our previous studies demonstrating that µ-opioid signaling releases endolysosomal iron stores to the cytoplasm and upregulates ferritin proteins in cortical neurons (Nash et al. 2019), these studies show that µ-opioid receptors recruit the endolysosomal iron transporter DMT1 to control neuronal iron metabolism and drive later upregulation of ferritin heavy chain.

## Discussion

Several recent studies suggest that opioid signaling may contribute to structural and functional deficits in central nervous system neurons by modulating neuronal iron metabolism, as reviewed in (Nash et al. 2021). We built from this foundation to better understand the molecular players involved and identified the endolysosomal iron transporter DMT1 as a key component of this process. Here, we discuss the DMT1 isoforms most likely involved in morphine’s regulation of iron homeostasis in neurons, how opioid regulation of DMT1 might manifest in molecular and cellular pathways, and how our results might fit more broadly into endolysosomal pathophysiology and relevant neurologic diseases.

DMT1 isoforms have a similar ability to transport iron (Mackenzie et al. 2007) but are often located in different cellular regions. For example, studies in non-neuronal cells report that DMT1B-IRE isoforms are enriched in recycling endosomes near the plasma membrane, while DMT1B+IRE is located either on the cell membrane or on endolysosomes near the nucleus (Tabuchi et al. 2002; Lam-Yuk-Tseung and Gros 2006). Likewise, a study in rat hippocampal neurons reported robust expression of DMT1B+IRE that colocalized with LAMP1-expressing endolysosomes and not markers of early endosomes (Pelizzoni et al. 2012). These findings position DMT1-IRE to regulate transferrin-mediated iron uptake (Gammella et al. 2017), and DMT1+IRE to regulate iron stores from the breakdown of cellular machinery in lysosomes. In this context, our results suggest that morphine can regulate DMT1+IRE on LAMP1 expressing endolysosomes, since morphine significantly reduces iron content in these organelles and adjacent compartments upregulate FHC protein at later timepoints (Nash et al. 2019). However, morphine also modestly increased FHC levels in more distal processes of the same neurons (Nash et al. 2019), suggesting that it may also regulate DMT1-IRE on recycling endosomes. Thus, morphine and other µ-opioid agonists may regulate iron efflux from both pools of endolysosomes via DMT1.

Since opioid receptor signaling drives iron release from endolysosomes (Nash et al. 2019; Halcrow et al. 2022a), downstream opioid signaling events may directly regulate DMT1 isoforms in cortical neurons. In this case, one of the most likely mechanisms involves µ-opioid receptor activation of nitric oxide synthase proteins (Rodriguez-Munoz and Garzon 2013). Nitric oxide molecules can directly s-nitrosylate several residues on DMT1, which significantly increases iron flux through the transporter (Liu et al. 2018). Nitric oxide can also nitrosylate other proteins that interact with and regulate DMT1, including the small GTPase dexras1 (Cheah et al. 2006). Nitrosylated dexras1 complexes with DMT1 via the scaffold protein acyl-CoA binding domain 3 (ACBD3), which promotes iron efflux from lysosomal stores through the transporter (White et al. 2016). However, these downstream pathways may be limited to neurons that express µ-opioid receptors. Several studies report strong receptor expression in cortical GABAergic interneurons (Taki et al. 2000; Ferezou et al. 2007), while others report lower expression in some excitatory neurons (Schmidt et al. 2003), often localized to excitatory synapses (Liao et al. 2005). This suggests that morphine may directly regulate DMT1 isoforms in both excitatory and inhibitory cortical neurons, though inhibitory neurons are likely to be more susceptible. These pathways must still be validated in cortical neurons.

Interestingly, opioids may also regulate endolysosomal iron stores by increasing the activity of local neuronal networks. µ-opioid agonists typically suppress firing from cortical GABAergic interneurons, which can disinhibit local excitatory neurons (Zieglgansberger et al. 1979; Vaughan et al. 1997; Jiang et al. 2021). Additionally, morphine can cause µ-opioid receptor expressing astrocytes to release glutamate onto nearby neurons, further exciting the local network (Nam et al. 2021). This increased network activity causes excitatory neurons to release endolysosomal iron stores to the cytoplasm via DMT1, and the newly freed iron can dampen excitability in hippocampal neurons via a process that suppresses NMDA receptor signaling (White et al. 2016). In this scenario, morphine could regulate iron metabolism in neurons that do not express µ-opioid receptors through its effects on local network activity. It is also possible that these molecular and cellular-level pathways could occur simultaneously, resulting in a widespread dysregulation or modulation of neuronal iron metabolism.

Morphine requires DMT1 to upregulate neuronal FHC, but we cannot exclude that opioid signaling regulates neuronal iron metabolism in other ways via additional endolysosomal conduits like two-pore channels (Halcrow et al. 2022a) and TRPML1 (Grimm et al. 2012). In a broader sense, there is a wide variety of endolysosomal proteins, channels, and transporters that work together to achieve iron storage, release, and homeostasis via different mechanisms (Ballabio and Bonifacino 2020). For example, morphine increases the pH of neuronal endolysosomes while these organelles export iron to the cytoplasm (Nash et al. 2019; Halcrow et al. 2022a), which is likely caused by DMT1’s proton-coupled transport of iron (Gunshin et al. 1997; Mackenzie et al. 2007). However, endolysosomal pH could also be regulated through other channels and transporters. Further, endolysosomal pH can be increased by other disease-relevant stimuli like select HIV proteins (Halcrow et al. 2022b, c), which highlights a potential point of convergence between distinct HIV- and opioid-related pathways. To add to this complexity, endolysosomes interact with other cellular components and participate in inter-organellar signaling networks that may also regulate endolysosomal iron storage and release to other organelles (Afghah et al. 2020). Therefore, it is possible that these layers of regulation and cellular communications systems could be dysregulated by morphine and other µ-opioid agonists, in addition to drivers of neurologic disorders like HIV proteins, neuroinflammation, and neurotoxic amyloid proteins.

Our work demonstrates that morphine requires the endolysosomal iron transporter DMT1 to regulate neuronal iron metabolism, which may drive neuronal deficits associated with cognitive impairment in several neurologic disorders. We also report a new set of molecular tools designed to improve the methodology for studying DMT1 isoforms in the future. These findings and tools could be used to identify new drug targets for conditions related to neuroHIV, opioid use disorders, and other neurologic disorders that present with dysregulated iron metabolism.

## Data Availability

All data generated or analyzed during this study are included in this article. Raw data and materials from this study are available from the corresponding author, Olimpia Meucci, upon reasonable request.

## References

[CR1] Afghah Z, Chen X, Geiger JD (2020). Role of endolysosomes and inter-organellar signaling in brain disease. Neurobiol Dis.

[CR2] Ballabio A, Bonifacino JS (2020). Lysosomes as dynamic regulators of cell and organismal homeostasis. Nat Rev Mol Cell Biol.

[CR3] Brewer GJ, Torricelli JR, Evege EK, Price PJ (1993). Optimized survival of hippocampal neurons in B27-supplemented Neurobasal, a new serum-free medium combination. J Neurosci Res.

[CR4] Chang HC, Bayeva M, Taiwo B, Palella FJ, Hope TJ, Ardehali H (2015). Short communication: high cellular iron levels are associated with increased HIV infection and replication. AIDS Res Hum Retroviruses.

[CR5] Cheah JH, Kim SF, Hester LD, Clancy KW, Patterson SE, Papadopoulos V, Snyder SH (2006). NMDA receptor-nitric oxide transmission mediates neuronal iron homeostasis via the GTPase Dexras1. Neuron.

[CR6] Fennema-Notestine C, Thornton-Wells TA, Hulgan T, Letendre S, Ellis RJ, Franklin DR Jr, Anderson AM, Heaton RK, Bloss CS, Grant I, Kallianpur AR, Group CS (2020). Iron-regulatory genes are associated with Neuroimaging measures in HIV infection. Brain Imaging Behav.

[CR7] Ferezou I, Hill EL, Cauli B, Gibelin N, Kaneko T, Rossier J, Lambolez B (2007). Extensive overlap of mu-opioid and nicotinic sensitivity in cortical interneurons. Cereb Cortex.

[CR8] Festa LK, Irollo E, Platt BJ, Tian Y, Floresco S, Meucci O (2020) CXCL12-induced rescue of cortical dendritic spines and cognitive flexibility. Elife 9 10.7554/eLife.49717 PMC7007222 31971513

[CR9] Gammella E, Buratti P, Cairo G, Recalcati S (2017). The transferrin receptor: the cellular iron gate. Metallomics.

[CR10] Gao G, Li J, Zhang Y, Chang YZ (2019). Cellular Iron Metabolism and Regulation. Adv Exp Med Biol.

[CR11] Grimm C, Hassan S, Wahl-Schott C, Biel M (2012). Role of TRPML and two-pore channels in endolysosomal cation homeostasis. J Pharmacol Exp Ther.

[CR12] Gruenheid S, Canonne-Hergaux F, Gauthier S, Hackam DJ, Grinstein S, Gros P (1999). The iron transport protein NRAMP2 is an integral membrane glycoprotein that colocalizes with transferrin in recycling endosomes. J Exp Med.

[CR13] Gunshin H, Mackenzie B, Berger UV, Gunshin Y, Romero MF, Boron WF, Nussberger S, Gollan JL, Hediger MA (1997). Cloning and characterization of a mammalian proton-coupled metal-ion transporter. Nature.

[CR14] Halcrow PW, Kumar N, Hao E, Khan N, Meucci O, Geiger JD (2022a) Mu opioid receptor-mediated release of endolysosome iron increases levels of mitochondrial iron, reactive oxygen species, and cell death. Neuroimmune Pharmacol Ther 2(1):19–35 10.1515/nipt-2022-0013 PMC10070011 37027339

[CR15] Halcrow PW, Kumar N, Quansah DNK, Baral A, Liang B, Geiger JD (2022b) Endolysosome Iron Chelation Inhibits HIV-1 Protein-Induced Endolysosome De-Acidification-Induced Increases in Mitochondrial Fragmentation, Mitophagy, and Cell Death. Cells 11 10.3390/cells11111811 PMC9180803 35681506

[CR16] Halcrow PW, Lakpa KL, Khan N, Afghah Z, Miller N, Datta G, Chen X, Geiger JD (2022). HIV-1 gp120-Induced Endolysosome de-Acidification Leads to Efflux of Endolysosome Iron, and Increases in Mitochondrial Iron and Reactive Oxygen Species. J Neuroimmune Pharmacol.

[CR17] Howitt J, Putz U, Lackovic J, Doan A, Dorstyn L, Cheng H, Yang B, Chan-Ling T, Silke J, Kumar S, Tan SS (2009). Divalent metal transporter 1 (DMT1) regulation by Ndfip1 prevents metal toxicity in human neurons. Proc Natl Acad Sci USA.

[CR18] Jiang C, Wang X, Le Q, Liu P, Liu C, Wang Z, He G, Zheng P, Wang F, Ma L (2021). Morphine coordinates SST and PV interneurons in the prelimbic cortex to disinhibit pyramidal neurons and enhance reward. Mol Psychiatry.

[CR19] Lam-Yuk-Tseung S, Gros P (2006). Distinct targeting and recycling properties of two isoforms of the iron transporter DMT1 (NRAMP2, Slc11A2). Biochemistry.

[CR20] Lane DJR, Ayton S, Bush AI (2018). Iron and Alzheimer's Disease: An Update on Emerging Mechanisms. J Alzheimers Dis.

[CR21] Liao D, Lin H, Law PY, Loh HH (2005). Mu-opioid receptors modulate the stability of dendritic spines. Proc Natl Acad Sci USA.

[CR22] Liu C, Zhang CW, Lo SQ, Ang ST, Chew KCM, Yu D, Chai BH, Tan B, Tsang F, Tai YK, Tan BWQ, Liang MC, Tan HT, Tang JY, Lai MKP, Chua JJE, Chung MCM, Khanna S, Lim KL, Soong TW (2018). S-Nitrosylation of Divalent Metal Transporter 1 Enhances Iron Uptake to Mediate Loss of Dopaminergic Neurons and Motoric Deficit. J Neurosci.

[CR23] Mackenzie B, Takanaga H, Hubert N, Rolfs A, Hediger MA (2007). Functional properties of multiple isoforms of human divalent metal-ion transporter 1 (DMT1). Biochem J.

[CR24] Mochizuki H, Choong CJ, Baba K (2020). Parkinson's disease and iron. J Neural Transm (vienna).

[CR25] Nam MH, Won W, Han KS, Lee CJ (2021). Signaling mechanisms of mu-opioid receptor (MOR) in the hippocampus: disinhibition versus astrocytic glutamate regulation. Cell Mol Life Sci.

[CR26] Nash B, Irollo E, Brandimarti R, Meucci O (2021). Opioid Modulation of Neuronal Iron and Potential Contributions to NeuroHIV. Methods Mol Biol.

[CR27] Nash B, Tarn K, Irollo E, Luchetta J, Festa L, Halcrow P, Datta G, Geiger JD, Meucci O (2019) Morphine-Induced Modulation of Endolysosomal Iron Mediates Upregulation of Ferritin Heavy Chain in Cortical Neurons. eNeuro 6 10.1523/ENEURO.0237-19.2019 PMC6675873 31300544

[CR28] Nimchinsky EA, Sabatini BL, Svoboda K (2002). Structure and function of dendritic spines. Annu Rev Physiol.

[CR29] Patton SM, Wang Q, Hulgan T, Connor JR, Jia P, Zhao Z, Letendre SL, Ellis RJ, Bush WS, Samuels DC, Franklin DR, Kaur H, Iudicello J, Grant I, Kallianpur AR (2017). Cerebrospinal fluid (CSF) biomarkers of iron status are associated with CSF viral load, antiretroviral therapy, and demographic factors in HIV-infected adults. Fluids Barriers CNS.

[CR30] Pelizzoni I, Zacchetti D, Smith CP, Grohovaz F, Codazzi F (2012). Expression of divalent metal transporter 1 in primary hippocampal neurons: reconsidering its role in non-transferrin-bound iron influx. J Neurochem.

[CR31] Pitcher J, Abt A, Myers J, Han R, Snyder M, Graziano A, Festa L, Kutzler M, Garcia F, Gao WJ, Fischer-Smith T, Rappaport J, Meucci O (2014). Neuronal ferritin heavy chain and drug abuse affect HIV-associated cognitive dysfunction. J Clin Invest.

[CR32] Porras CA, Rouault TA (2022) Iron Homeostasis in the CNS: An Overview of the Pathological Consequences of Iron Metabolism Disruption. Int J Mol Sci 23 10.3390/ijms23094490 PMC9104368 35562883

[CR33] Rodriguez-Munoz M, Garzon J (2013). Nitric oxide and zinc-mediated protein assemblies involved in mu opioid receptor signaling. Mol Neurobiol.

[CR34] Rouault TA (2013). Iron metabolism in the CNS: implications for neurodegenerative diseases. Nat Rev Neurosci.

[CR35] Santi C, Scimmi C, Sancineto L (2021) Ebselen and Analogues: Pharmacological Properties and Synthetic Strategies for Their Preparation. Molecules 26 10.3390/molecules26144230 PMC8306772 34299505

[CR36] Saylor D, Dickens AM, Sacktor N, Haughey N, Slusher B, Pletnikov M, Mankowski JL, Brown A, Volsky DJ, McArthur JC (2016). HIV-associated neurocognitive disorder–pathogenesis and prospects for treatment. Nat Rev Neurol.

[CR37] Schmidt P, Schmolke C, Musshoff F, Menzen M, Prohaska C, Madea B (2003). Area-specific increased density of mu-opioid receptor immunoreactive neurons in the cerebral cortex of drug-related fatalities. Forensic Sci Int.

[CR38] Sengupta R, Burbassi S, Shimizu S, Cappello S, Vallee RB, Rubin JB, Meucci O (2009). Morphine increases brain levels of ferritin heavy chain leading to inhibition of CXCR4-mediated survival signaling in neurons. J Neurosci.

[CR39] Shawki A, Knight PB, Maliken BD, Niespodzany EJ, Mackenzie B (2012). H(+)-coupled divalent metal-ion transporter-1: functional properties, physiological roles and therapeutics. Curr Top Membr.

[CR40] Singh N, Haldar S, Tripathi AK, Horback K, Wong J, Sharma D, Beserra A, Suda S, Anbalagan C, Dev S, Mukhopadhyay CK, Singh A (2014). Brain iron homeostasis: from molecular mechanisms to clinical significance and therapeutic opportunities. Antioxid Redox Signal.

[CR41] Skjorringe T, Burkhart A, Johnsen KB, Moos T (2015). Divalent metal transporter 1 (DMT1) in the brain: implications for a role in iron transport at the blood-brain barrier, and neuronal and glial pathology. Front Mol Neurosci.

[CR42] Tabuchi M, Tanaka N, Nishida-Kitayama J, Ohno H, Kishi F (2002). Alternative splicing regulates the subcellular localization of divalent metal transporter 1 isoforms. Mol Biol Cell.

[CR43] Tabuchi M, Yoshimori T, Yamaguchi K, Yoshida T, Kishi F (2000). Human NRAMP2/DMT1, which mediates iron transport across endosomal membranes, is localized to late endosomes and lysosomes in HEp-2 cells. J Biol Chem.

[CR44] Taki K, Kaneko T, Mizuno N (2000). A group of cortical interneurons expressing mu-opioid receptor-like immunoreactivity: a double immunofluorescence study in the rat cerebral cortex. Neuroscience.

[CR45] Torti FM, Torti SV (2002). Regulation of ferritin genes and protein. Blood.

[CR46] Vaughan CW, Ingram SL, Connor MA, Christie MJ (1997). How opioids inhibit GABA-mediated neurotransmission. Nature.

[CR47] Wetli HA, Buckett PD, Wessling-Resnick M (2006). Small-molecule screening identifies the selanazal drug ebselen as a potent inhibitor of DMT1-mediated iron uptake. Chem Biol.

[CR48] White RS, Bhattacharya AK, Chen Y, Byrd M, McMullen MF, Siegel SJ, Carlson GC, Kim SF (2016). Lysosomal iron modulates NMDA receptor-mediated excitation via small GTPase, Dexras1. Mol Brain.

[CR49] Yanatori I, Kishi F (2019). DMT1 and iron transport. Free Radic Biol Med.

[CR50] Zieglgansberger W, French ED, Siggins GR, Bloom FE (1979). Opioid peptides may excite hippocampal pyramidal neurons by inhibiting adjacent inhibitory interneurons. Science.

